# High-performance wireless powering for peripheral nerve neuromodulation systems

**DOI:** 10.1371/journal.pone.0186698

**Published:** 2017-10-24

**Authors:** Yuji Tanabe, John S. Ho, Jiayin Liu, Song-Yan Liao, Zhe Zhen, Stephanie Hsu, Chika Shuto, Zi-Yi Zhu, Andrew Ma, Christopher Vassos, Peter Chen, Hung Fat Tse, Ada S. Y. Poon

**Affiliations:** 1 Department of Electrical Engineering, Stanford University, Stanford, California 94305, United States of America; 2 Department of Electrical and Computer Engineering, National University of Singapore, Singapore 117583, Singapore; 3 Center for Innovation and Strategic Collaboration, St. Jude Medical, Inc., Orange County, California 92618, United States of America; 4 Department of Medicine, University of Hong Kong, Hong Kong, China; 5 Hong Kong-Guangdong Joint Laboratory on Stem Cell and Regenerative Medicine, University of Hong Kong, Hong Kong, China; Beijing University of Posts and Telecommunications, CHINA

## Abstract

Neuromodulation of peripheral nerves with bioelectronic devices is a promising approach for treating a wide range of disorders. Wireless powering could enable long-term operation of these devices, but achieving high performance for miniaturized and deeply placed devices remains a technological challenge. We report the miniaturized integration of a wireless powering system in soft neuromodulation device (15 mm length, 2.7 mm diameter) and demonstrate high performance (about 10%) during *in vivo* wireless stimulation of the vagus nerve in a porcine animal model. The increased performance is enabled by the generation of a focused and circularly polarized field that enhances efficiency and provides immunity to polarization misalignment. These performance characteristics establish the clinical potential of wireless powering for emerging therapies based on neuromodulation.

## Introduction

Targeted modulation of peripheral nerves with a bioelectronic device is a clinical treatment modality for hypertension [1], depression [2], pain [3], and inflammation [4], and is anticipated to be effective to many other disorders [5]. Achieving lasting therapeutic effect requires chronic operation of the devices, but long-term powering of bioelectronics in the human body remains a major technological challenge. Most commercial devices rely on batteries, which are bulky, have limited lifetimes, and require periodic surgical replacement. Although energy harvesting strategies based on biopotentials [6], glucose [7], or physiological motion [8] have been developed, existing technologies yield power densities too low for a miniaturized device.

Wireless powering has been extensively studied as a potential approach to achieve high power densities and long operational lifetimes in bioelectronic systems [9–25]. The most widely used systems are based on coils coupled inductively in the near-field where interactions with tissue are suppressed by a predominant magnetic field [9–19]. While high-efficiency systems have been developed for large, centimeter-diameter devices implanted near the surface of the body, their integration into a miniaturized device is constrained by weak coupling at depths greater than the dimensions of the structure, a consequence of the evanescent decay of the near-field. For clinical neuromodulation, existing systems use solenoidal coils (< 4 mm^2^ cross-section area, >18 mm length) with millimeter diameters to accommodate implantation on peripheral nerves, but the reported transfer efficiencies are less than 1% [26] beyond superficial depths in tissue. An alternative approach is to operate at low gigahertz frequencies where the wavelength in tissue is comparable to the distance of transfer [20–25]. Power transfer in this regime, termed the midfield, occurs through propagating fields without intrinsic evanescent decay, but must overcome challenges in tissue absorption, directionality of energy transport, and polarization alignment. Both approaches have been used to power commercial neuromodulation devices, including peripheral nerve stimulators for pain (StimRouter, Bioness, Valencia, CA, USA; Freedom, StimWave Technologies, Pompano Beach, FL, USA) [27, 28] and for overactive bladder (BlueWind Medical, Herzliya, Israel) [29]. However, owing to limitations in the wireless powering system, these devices are large (>20 mm in length) and require careful alignment of the external transmitter for efficient operation. Other wireless powering approaches based on capacitive [30], optical [31], or ultrasonic [32] transfer mechanisms have also been recently explored, although these systems have yet to be deployed in large animals.

We have previously developed a wireless powering method based in the midfield regime in which the field pattern within the body is shaped to enhance performance [22, 25]. Here we report the integration of this system in a miniaturized neuromodulation device and demonstrate high performance wireless powering during *in vivo* modulation of the vagus nerve in a large animal model. The high performance is enabled by the design of a transmitter that controls both the field shape and polarization. By modulating the phase on the body surface, the generated field is focused and circularly polarized to enhance efficiency and provide immunity to polarization misalignment. We demonstrate wireless powering of a neuromodulation device that integrates a subwavelength dipole, power harvesting circuitry, and tripolar stimulation electrodes within a 15 mm length (<2 mm width) neural cuff, representing significant miniaturization [27–29]. We characterize the performance of the system and illustrate *in vivo* operation by wirelessly regulating heart rate and blood pressure in a porcine animal model.

## System design

Operation of the wireless powering system in the midfield regime involves selection of the system frequency such that the wavelength in tissue is comparable to the distance of separation. We designed the system to operate at 2.4 GHz within the industrial, scientific, and radio-frequency (ISM) band, yielding a wavelength of about 1.7 cm in muscle tissue. By tailoring the structure of the field source, the three-dimensional pattern and polarization of the field in tissue can be shaped at depths comparable to or greater than the wavelength (see Methods, Transmitter Design).

**Fig 1:**
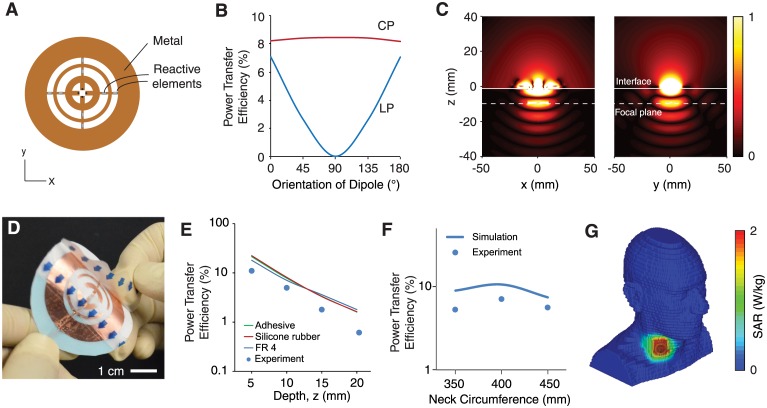
Conformal wireless powering transmitter. A: Transmitter consisting of concentric metal rings. Adjacent rings are connected and loaded with reactive elements along the *x* and *y* axes to generate circularly polarized (CP) field or linearly polarized (LP) field. B: Simulated power transfer efficiency to a 20-mm straight dipole as a function of its orientation. The transmitter is placed above a tissue medium. The dipole is 10 mm deep in tissue. C: Contour plot of the electric field intensity generated by reactive elements along the *x* axis. D: Photograph of the transmitter. E: Numerically simulated and measured power transfer efficiency to a 20-mm long dipole as a function of its depth in tissue. F: Numerically simulated and measured power transfer efficiency over a curved surface. G: Simulated specific absorption ratio (SAR) using a human voxel model. Measured efficiency is recorded in saline solution. Simulations and measurements are performed at 2.4 GHz.

Fig 1A shows the design of wireless powering source consisting of reactively loaded rings (3 cm diameter) laser-cut from copper film and encapsulated in soft silicone (Fig 1D). The structure supports two sets of orthogonal, semicircular ring-like surface currents whose coupling can be tailored by the choice of reactive element [33]. We used a numerical optimization procedure [25] to set the phase between the current to *π*/2 such that the radiated field is circularly polarized. Owing to the rotation of the electric field, the wireless powering performance to polarization alignment (rotation in the transverse plane), as shown by the simulated results in Fig 1B. Interference between the radiation also results in moderate focusing of the field (Fig 1C), providing enhancement of performance when the source is placed over the device. The design of the structure is based on the Babinet complement of the source reported in Ref. [34] to generate a dominant electric, rather than magnetic, response.

**Fig 2:**
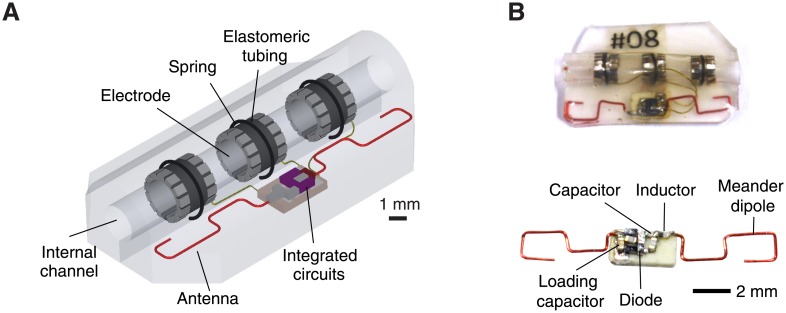
Design of wireless cuff electrodes. A: Schematic diagram of a wireless cuff. The cuff consists of an array of electrodes, a meandered antenna, integrated circuits, and an inner channel wrapped around a nerve. B: Photos of the wireless cuff, and the embedded integrated circuits and antenna.

The device extracts energy from the radio-frequency field through a miniaturized (2 cm long) dipole antenna (Fig 2A). In contrast with near-field systems, the dipole antenna can be efficient because of the similar amplitudes of the electric and magnetic field in the midfield region. The small diameter of the structure is important to enable minimally invasive implantation and to accommodate compact tissue around peripheral nerves.

The wireless neuromodulation device consists of the dipole antenna, matching network, energy harvesting circuit, energy-storage capacitor, and tripolar electrodes integrated onto a soft elastomeric substrate (Fig 2B). The electrodes are fabricated from shape-memory alloy (Pt-coated NiTi alloy) and transitions from an open to closed state upon heating from room to body temperature. In the closed state, the device is mechanically secure around minimum 1.1-cm diameter nerve without requiring sutures or adhesives, minimizing damage to the nerve and surrounding tissues. The entire device weighs about 0.3 g and is 3 mm at the thickest point, ensuring minimal mechanical loading of the surrounding tissues.

## Results

### Wireless powering performance

We first characterized the performance of the wireless powering system in saline. At a depth of 1-cm heterogeneous tissue, the efficiency of power transfer to a 2-cm length dipole was about 8% (Fig 1E) in both measurements and simulations (Methods, Efficiency Measurements). The performance of the system is the same whether the source is fabricated on a conventional microwave substrate (FR4) or soft silicone (Fig 1E), suggesting that substrate losses play a relatively negligible role in performance.

Conformal placement of the source on non-planar interfaces requires that the system maintain high performance under curvature. We measured a power transfer efficiency of about 10% for typical circumference of the neck (40 cm). Measurements and simulations show that variation of the neck circumference to 35 cm and 45 cm does not significantly affect the performance, demonstrating robustness under moderate curvature.

**Fig 3:**
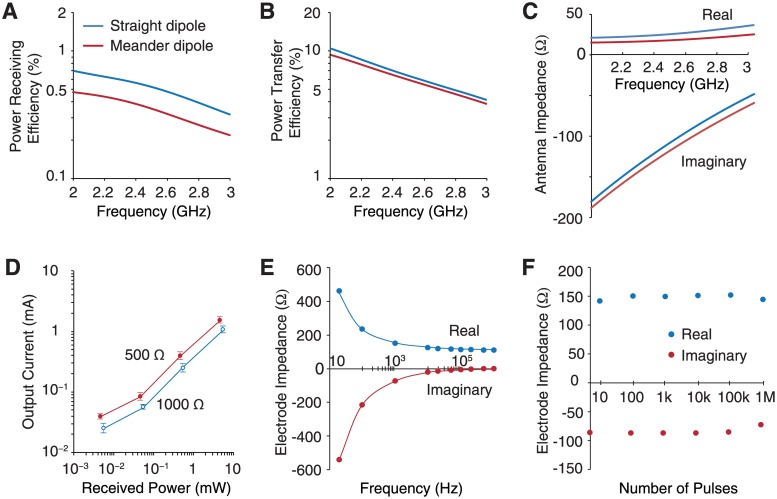
Performance and electrical properties of wireless cuff electrodes. A—C: Simulated performance of a 20-mm long straight dipole versus a 14-mm meandered dipole as a function of operating frequency. The dipoles are placed 10 mm deep in a tissue medium. D: Efficiency of the integrated circuits in converting radio-frequency power into current across loading impedances of 500 Ω and 1000 Ω (*n* = 2, minimum, mean, maximum). E: Real and imaginary parts of electrode impedance spectrum recorded in saline solution. F: Electrode impedance at 1 kHz after applying pulse train. Pulse train parameters, 25 Hz, 1 V amplitude, 500 *μ*s pulse width.

The meandered dipole antenna with wire length of *λ*/2 is used for the receiver structure in order to minimize device dimensions. Because the operating regime is not in the far-field, the design circumvents limitations on the meandered dipole performance relative to that of the straight dipole. Measurements and simulation show that meandered structure achieves similar power transfer efficiency (Fig 3A and 3B) and antenna impedance (Fig 3C) as compared to straight dipole despite the decreased length.

The safety characteristics of the system were studied using numerical simulations with a human body model. Using the more stringent guidelines for uncontrolled environments, the maximum specific absorption rate (SAR) is 2 W/kg, averaged over 10 g of tissue [35]. For the transmitter placed on the human neck above the vagus nerve (Fig 1G), the maximum output power consistent with this threshold is 182 mW averaged over the pulse duration cycle. The system achieves power transfer efficiencies from 5% to 10%, corresponding to a time-averaged power of 9.1 mW to 18.2 mW under the safety guidelines. These power levels substantially exceeds requirements for clinical vagus nerve modulation, which dissipate direct-current power on the order of 10 to 100 *μ*W averaged over the pulse cycle [36].

### Circuit and electrode performance

We next characterized the ability of the device to generate the range of output pulses required for neuromodulation. Under the power levels delivered by the wireless powering system, the device meets a wide range of stimulation requirements: the output current can range from 10 *μ*A to 2 mA (Fig 3D) for load impedances ranging from 500 Ω to 1000 Ω. Saline measurements yield a lower bound of 150 Ω at 1 kHz on electrode impedance that can be achieved *in vivo* (Fig 3E). The electrodes exhibit excellent stability, maintaining nearly constant impedance over one million pulse cycles (1 V, 25 Hz, monophasic, 500 *μ*s pulse width) (Fig 3F). The efficiency of the harvesting circuit is greater than 20% when sourcing a current of 1 mA to a 1000 Ω load (typical of chronically implanted electrodes and biomedical integrated circuits, but decreases to 13% for a 150 Ω load. Further optimization of the harvesting circuit can yield higher conversion efficiencies, although the present performance is already sufficient for neuromodulation.

### *In vivo* experiments

**Fig 4:**
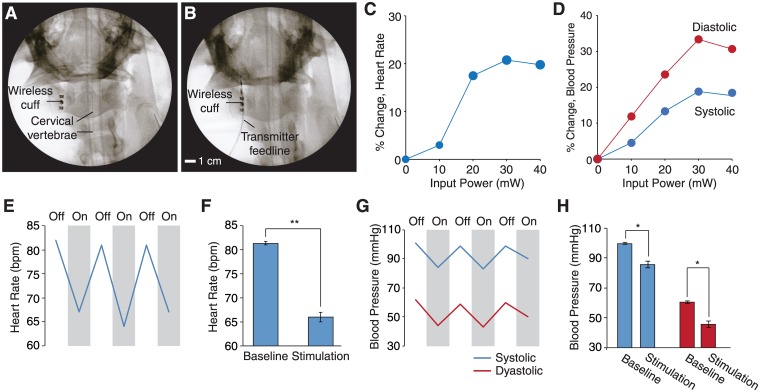
Wireless right vagus nerve stimulation in vivo. A—B: Fluoroscopy images of the implanted wireless cuff and transmitter on skin. C—D: Percentage change in heart rate and blood pressure versus input power to the transmitter. E: Heart rate during stimulation trials. Stimulation is off for about 30 sec before each stimulation to allow heart rate to return to baseline. F: Trial means (P = 0.0033, paired T-test). G: Blood pressure during stimulation trials. H: Trial means for systolic (P = 0.031, paired T-test) and diastolic (P = 0.026, paired T-test) blood pressure. Stimulation parameters, 20 Hz, 500 *μ*s pulse width, and 35 mW input power. Error bars are s.e.m.

To illustrate operation of the system *in vivo*, we wirelessly modulated the vagus nerve in anesthetized pigs (*n* = 3, female, adult 40–45 kg). Following an incision in the right neck, the cuff device (2.7 mm inner diameter, 15 mm length, 1 mm electrode width, 2 mm electrode spacing) was attached to the right cervical vagus nerve (Fig 4A) and implanted by closing the incision using adhesive dressing. Projection radiography shows the wireless powering source attached to the skin surface approximately 1.5 cm above the device (Fig 4B). We monitored the heart rate by surface electrocardiogram, and blood pressure by an intra-arterial line over the femoral artery.

Monophasic pulses were generated across the electrodes by pulsed excitation of the wireless powering source (20 Hz and 500 *μ*s pulse width). Monitoring of the heart rate and blood pressure revealed depression of heart rate and blood pressure with magnitude increasing with stimulation amplitude up to a saturation threshold. Peak change in heart rate (20% decrease) was achieved at an 20 mW output power level from the source (Fig 4C), and at 30 mW for diastolic (30% decrease) and systolic (20% decrease) blood pressure (Fig 4D). Beyond this threshold, no further reduction in heart rate and blood pressure was observed.

**Fig 5:**
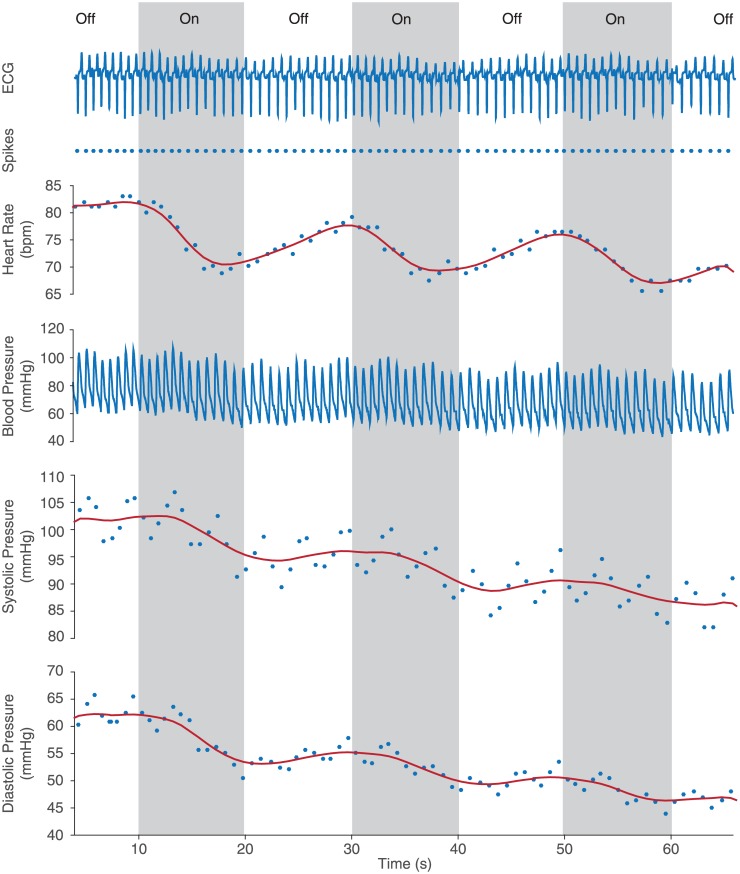
Real-time *in vivo* responses. First row records ECG waveform from lead III. Second row shows spikes extracted from the ECG waveform. Third row shows heart rate oscillation during 10-s alternating on-off stimulation. Forth row records blood pressure waveform. Fifth and sixth rows show the decrease in systolic and diastolic pressures during the 10-s alternating on-off stimulation.

We demonstrated reversibility of the induced heart rate and blood pressure depression by alternating between on and off stimulation periods. Stimulation for 30 s followed by an off duration of 30 s showed consistent restoration of heart rate and blood pressure to baseline values over multiple trials (Fig 4E–4H). The response dynamics were also studied by alternating between 10-s stimulation periods. Both heart rate and blood pressure exhibit rapid (<2 s) response to the stimulus but slower recovery once the stimulation pulses cease (Fig 5). These performance results demonstrate that the wireless powering is efficient and robust across a range of complex and heterogenous tissue environments typical of those encountered in clinical neuromodulation.

## Conclusion

We have demonstrated the miniaturized integration of wireless powering system in a neuromodulation device and demonstrated high performance operation *in vivo* in a porcine animal model. The system is based on operation in the electromagnetic midfield regime and obtains high efficiency and robustness through optimization of the field shape and polarization. The designed electromagnetic structures are thin and flat, which enables their integration into soft and conformal biomedical devices. Large animal experiments demonstrate the ability of the system wirelessly control heart rate and blood pressure by modulating neural activity in the vagus nerve using a wireless cuff-type stimulator.

Because our approach enables substantially smaller and lighter devices, the wireless powering system could enable access to key neural targets in deep and compact tissue regions. Potential peripheral nerves include the sacral nerve for urinary control [37], occipital nerve for pain control [38], and spinal cord for restoring voluntary control of locomotion [39]. The high efficiency of the system also opens opportunities for optical neuromodulation approaches, such as optogenetics [40, 41] using micron-scale light-emitting diodes (LEDs) [42]. These capabilities could address key challenges in powering the miniaturized, wireless devices expected to play a central role in the emerging field of bioelectronic medicines.

## Methods

### Transmitter design

Wireless power transfer was simulated using a commercial finite-difference time domain solver (CST Microwave Suite). The transmitter was modeled by three concentric copper rings of variable trace width, yielding optimal diameters and traces widths of 8.25 mm and 1.7 mm for the inner ring, 14.9 mm and 0.854 mm for the middle ring, and 27.2 mm and 5.62 mm for the outer ring. The thickness of the copper was set to 30 *μ*m on a FR4 substrate. The structure was excited across the center with single port to generate linearly polarization or two ports with a *π*/2 offset to generate circularly polarization, separated vertically by 1 mm gap. To calculate the impedance values of the passive elements, a multiport simulation of the structure was performed by placing the receiver in a multilayered medium composed of air, skin (2 mm) and muscle (74 mm) at varying depths. The values of the reactive elements required to generate focusing were obtained by the numerical optimization procedure as described in Ref. [25]. Ideally, the efficiency from the linearly polarized and the circularly polarized transmitters should be the same at received dipole orientation of 0° and 180°. The slight discrepancy between the two efficiencies in Fig 1B is due to the discrete-port effect.

### Fabrication

Two different approaches were used to fabricate the transmitter: The first approach was based on an ultrathin FR4 microwave substrate. A 25-*μ*m thick copper foil was laser cut and temporarily connected with polyimide taps. Reactive elements and feed cable were soldered on copper foil. The temporary polyimide tapes were removed. A sheet of 0.25-mm thick unvulcanized silicone was attached to a vulcanized silicone, and cut to size. Another 0.25-mm thick vulcanized silicone was cut to size. Copper traces with reactive elements were centered on vulcanized silicone, then a double-layer silicone (unvulcanized side down) was placed on top of the copper trace. The assembly was cured in 130°C oven for 2 hours. Weights were placed on top of silicone during curing process to secure bonding. The gap between silicone sheets and feeding cable was filled with RTV silicone.

In the second approach, the conductive trace was laser cut from a 0.001-inch copper foil with temporary connections. The connections were cut off after reactive elements and feed cable were soldered on the trace. The copper traced sub-assembly was placed on Tegaderm (3M Company) transparent dressing and then covered by a 25-*μ*m polyurethane film with acrylate adhesive. Fig 1D shows the fabricated transmitter.

### Efficiency measurements

The efficiency measurement in Fig 1F was done in three steps. In the first step, we found the optimal matching network to the dipole antenna. The dipole was connected to a matching network which was an inductor. The matching network was connected to an energy harvesting circuit. The output of the network was connected to a reference load of *Z*_*L*_ = 1 kΩ, which is typical of chronically implanted electrodes and many classes of biomedical integrated circuits. A pair of insulated, twisted cables was attached to the two terminals of the load resistor. The completed device is shown in Fig 2B. The device was encapsulated by epoxy glue (GC Electronics 10-114) and was submerged in phosphate buffered saline solution (BP399-1 Fisher BioReagents). The voltage across the load was measured by an oscilloscope (Tektronix TBS 1102B) via the pair of twisted cables. The transmitter was connected to a power amplifier (Mini-Circuits ZHL-5W-2G-S+). To ease the measurement, the transmitter was fabricated on FR-4 substrate. By varying the value of the inductor, we recorded the output voltage and found out the optimal inductor value which was 10 nH. The setup is shown in S4B Fig.

In the second step, we characterized the non-linearity of the energy harvesting circuit. The setup is shown in S4C Fig. The input of the energy harvesting circuit was connected to a vector network analyzer (Agilent E5072A). By measuring the reflection *S*_11_, we obtained the net input power to the circuit. The output of the circuit was connected to a variable load *Z*_*L*_. The voltage across the load was measured by the oscilloscope to compute the output power. By varying the load impedance, we tabulated the relationship between the input power and the output power of the energy harvesting circuit.

In the last step, we measured the power transfer efficiency between the transmitter and the dipole. A continuous-wave signal at 2.247 GHz with output power ranging from 0.15 W to 5.82 W was fed to the transmitter. Output power from the power amplifier was measured by a power sensor (Mini-Circuits PWR-6G+). The transmitter was positioned right above the liquid solution. The reflection *S*_11_ from the air-solution interface was measured to be −8.14 dB. We could then obtain the net input power to the transmitter. The encapsulated receiver (dipole, matching network, and load) was submerged in the liquid solution. Output voltage across the load was measured by the oscilloscope. From the table obtained in the second step, we calibrated out the efficiency of the energy harvesting circuit and obtained the link efficiency.

### Electrode measurements

The impedance spectrum in Fig 3E was measured with the cuff electrodes fully submerged in the phosphate buffered saline solution. The impedance was measured by an LCR meter (Agilent 4284A) at room temperature (S5A Fig). Then, a 25-Hz stimulation pulse train of 500-*μ*s pulse width and 1-V amplitude was delivered to the electrodes using a function/arbitrary waveform generator (BK precision 4052) shown in S5B Fig. After delivering certain number of pulses, we used the LCR meter to measure the electrode impedance at 1 kHz before resuming the stimulation pulse train.

### In vivo experiments

Three female adult pigs (age 9–12 months; and weight approximately 40–45 kg) were used. During the surgical procedure, all animals were anesthetized with tiletamine and zolezepam (Zoletil 20 mg/kg intramuscularly). Endotracheal intubation was performed, and anaesthesia was maintained with isoflurane (1.5% to 2.0%) and oxygen while the animals were mechanically ventilated. A sheath was inserted into the femoral artery and connected to a transducer to measure arterial blood pressure. Surface electrodes were placed on the legs of the animal to record the electrocardiogram for measurement of heart rate. The surface electrocardiogram and arterial blood pressure signals were digitally recorded using CardioLab (GE Healthcare). Following a 5-cm incision over the right neck region, the right cervical vagus nerve was exposed by careful dissection. The wireless cuff stimulator was placed around the right vagus nerve. After apposition of the incision using the adhesive dressing, the transmitter was placed outside the body on the skin to perform stimulation experiment. The animals were euthanized after the experiment. These experiments were approved by the Committee on the Use of Live Animals in Teaching and Research at the University of Hong Kong.

## Supporting information

S1 Fig Wireless transmitter design. A: Phase-control surface consisting of reactively loaded concentric rings. B: Wireless transmitter based on its Babinet complement.
(EPS)

S2 Fig Field pattern generated by the phase-control surface and the Babinet-complement structure. The field generated by the Babinet-complement structure has a shallow focus with electric field in the illustrated plane.
(EPS)

S3 Fig Electric field generated by electrodes. The field is shown for two alternating tripolar stimulation configurations.
(EPS)

S4 Fig Experimental setup for device and power transfer characterization. A: Circuit schematic of the device and layout on a printed circuit board. Twisted cables are used to probe the direct-current voltage generated over the load. B: Schematic of the wireless characterization setup in saline. C: Schematic of the circuit characterization setup. The radio-frequency signal is directly applied across the input terminals.
(EPS)

S5 Fig Experimental setup for electrode characterization. A: Impedance measurement in saline. B: Electrical pulse delivery in saline.
(EPS)

S1 File ARRIVE checklist. Reporting of *in vivo* experiments guidelines.
(PDF)
